# NKT Cell Stimulation with α-Galactosylceramide Results in a Block of Th17 Differentiation after Intranasal Immunization in Mice

**DOI:** 10.1371/journal.pone.0030382

**Published:** 2012-01-26

**Authors:** Beata M. Zygmunt, Sebastian F. Weissmann, Carlos A. Guzman

**Affiliations:** Department of Vaccinology and Applied Microbiology, Helmholtz Centre for Infection Research, Braunschweig, Germany; University Hospital Freiburg, Germany

## Abstract

In a previous study we demonstrated that intranasal (i.n.) vaccination promotes a Th17 biased immune response. Here, we show that co-administration of a pegylated derivative of α-galactosylceramide (αGCPEG) with an antigen, even in the presence of Th17-polarizing compounds, results in a strong blocking of Th17 differentiation. Additional studies demonstrated that this phenomenon is specifically dependent on soluble factors, like IL-4 and IFNγ, which are produced by NKT cells. Even NK1.1 negative NKT cells, which by themselves produce IL-17A, are able to block Th17 differentiation. It follows that the use of αGCPEG as adjuvant would enable to tailor Th17 responses, according to the specific clinical needs. This knowledge expands our understanding of the role played by NKT cells in overall control of the cytokine microenvironment, as well as in the overall shaping of adaptive immune responses.

## Introduction

Natural Killer T (NKT) cells are a unique cell population, which shares the features of cells from the adaptive and innate immune systems [1], [2]. Like T cells, they express on their surface a T cell receptor (TCR). However, the restriction of antigenic specificity by this TCR makes them more similar to cells belonging to the innate immune system. The most studied NKT cell subpopulation in mice, invariant NKT (iNKT) cells, express an invariant TCR encoded by Vα14 rearranged to Jα18, paired with β chains with limited heterogeneity [1], [2]. These cells recognize exogenous and endogenous lipids presented on the CD1d molecule. After recognition of an antigen, NKT cells rapidly produce different cytokines (e.g. IL-4 and IFNγ), thereby becoming potent regulators of the immune response [1], [2]. It was shown that activation of this cell subset leads to Th2 biased immune response [3]. This Th2 bias was demonstrated to play a role in the protection from experimental autoimmune encephalomyelitis (EAE) conferred by NKT cells [4], [5]. This autoimmune disease was considered until recently to be mediated by Th1 cells. However, the discovery of a new Th lineage, the Th17, brought new light on our understanding of the underlying mechanisms for this pathological condition. Currently, it is broadly accepted that Th17 cells, characterized by expression of IL-17A, are responsible for the development of EAE and there are numerous studies showing that blockage of the Th17 immune response leads to prevention of EAE development [6]. These results provided indirect evidence suggesting that NKT cells may be responsible for blockage of Th17 immune responses, as recent studies seems to further support [7]. NKT cells were also shown to regulate experimental autoimmune uveitis, through inhibition of Th17 differentiation [8].

However, it remains to be established if these properties of NKT cells can be exploited for medical applications and to which extent. On the other hand, the fact that NKT cells contribute to block Th17 differentiation seems especially intriguing, particularly taking under account that a NK1.1 negative subpopulation of NKT cells has been described, which secrete IL-17A upon stimulation [9], [10], [11]. One of the territories in which these cells are well-represented is the respiratory track, where the produced IL-17A is involved in airway neutrophilia.

One of the antigens recognized by iNKT cells is α-galactosylceramide. This glycolipid exhibits potent adjuvant properties by inducing full maturation of dendritic cells (DC) *in vivo* in a NKT cell dependent way [12]. This molecule can be also exploited as mucosal adjuvant, leading to potent cellular and humoral immune responses when administered by intranasal (i.n.) route [13]. Previous work from our group led to the development of a pegylated derivative of α-galactosylceramide (αGCPEG), which shows improved physicochemical and biological properties [14].

In a previous study we showed that i.n. immunization leads to the specific stimulation of Th17 immune responses, and that this is an intrinsic feature of this route of immunization independently of the adjuvant used [15]. Here, we demonstrate that co-administration of αGCPEG with an antigen results in a blockage of Th17 differentiation after i.n. immunization, and that this phenomenon is dependent on NKT cells. Interestingly, also NK1.1 negative NKT cells, which by themselves produce IL-17A, can block Th17 differentiation. This inhibition is mediated by soluble factors, playing IL-4 and IFNγ an important role in this process. Thus, our results provide the proof of concept for the usefulness of αGCPEG to specifically prevent or block Th17 cells stimulation when administered as stand-by-itself vaccine adjuvant or in combination with other compounds, when dictated by the specific medical needs.

## Materials and Methods

### Mice

C57BL/6 mice were purchased from Harlan (Borchen, Germany) and were used at the age 8 to 16 weeks. The OT-II (expressing the OVA323–339/Ab-specific TCR) and Jα281 knock out (KO) animals on C57BL/6 background were breed under specific pathogen free conditions at the Helmholtz Centre for Infection Research and the Max Planck Institute for Infection Biology animal facilities, respectively.

### Ethics Statement

This manuscript has not include any data generated using samples derived from humans or non-human primates. All animal experiments have been performed in accordance with institutional guidelines and have been approved by the local government (permission number 33.11.42502-04-017/08 and 33.11.42502-04-104/07/2007 from the “Niedersächsisches Landesamt für Verbraucherschutz und Lebensmittelsicherheit”).

### Antibodies

The following antibodies have been used: anti-IL-17A-APC, anti-IL-17A-PE, anti-IL-17F-APC, anti-IL-22-PerCP-eF710, anti-CD4-PE-Cy7, anti-CD44-APC, anti-NK1.1-APC, anti-IL-4, anti-IL-10 and anti-IFNγ from eBioscience; anti-CD62L-FITC, anti-CD8-PE, anti-B220-PE, anti-CD11c-PE, anti-CD11b-PE, anti-DX5-PE from BD Bioscience. The CD1d tetramer was purchased from Proimmune and loaded with αGCPEG.

### Immunization

The animals were immunized by i.n. route on day 0, 14 and 21 with 50 µg of ovalbumin (OVA; Sigma, purity grade VIII) alone or co-administered with either 5 µg αGCPEG, 10 µg lipopolysaccharide (LPS), 1 µg S-[2,3-bispalmitoyiloxy-(2R)-propyl]-R-cysteinyl-amido-monomethoxy polyethylene glycol (bisBPPcysPEG), 10 µg cholera toxin B subunit (CTB), 200 µg Curdlan, 20 µg CpG, 7.5 µg ISCOM, or 2.5 µg cdiGMP. The animals were sacrificed on day 42. In experiments with Jα281 KO mice, the animals were immunized on day 0 and 14 and sacrificed on day 34. Control animals received phosphate buffered saline (PBS).

### Cell isolation

In all the experiments mice were euthanized by CO_2_ inhalation. Lymphoid organs were dissected and single-cell suspensions were obtained by mincing organs through a 100 µm nylon mesh. Erythrocytes were lyzed by using the ACK buffer.

### Flow cytometry and cell sorting

Cells were stained with the antibodies and data acquisition was performed using a FACSCanto or FACSLSRII (BD Biosciences). Data were analyzed using FACSDiva (BD Bioscience) and FlowJo (Tree Star, Ashland, OR). Cells were sorted on a MOFlo (Dako Cytomation) or FACSAria (BD Bioscience). In some experiments, before sorting splenocytes were depleted from B cells using PanB beads (Dynal), according to the manufacturer's protocol.

### *In vitro* proliferation

Naïve cells (CD4^+^, CD44^low^, CD62L^high^) from OTII mice were sorted and stained with carboxyfluorescein diacetate succinimydyl ester (CFSE). These cells were then co-cultured with bone marrow derived DC (BMDC) at a 20∶1 ratio, in the presence of antigen (OVA peptide 323–339). NKT cells (NK1.1^+^, CD8^−^, B220^−^, CD11c^−^, CD11b^−^, DX5^−^) were sorted from spleen of C57BL/6 mice and added to some cultures at a 1∶5 T cells ratio. For experiments where NK1.1 negative and positive NKT cells were used, cells were sorted using anti-NK1.1 antibody and CD1d tetramer. Th17 inducing conditions were obtained by adding to the culture IL-6 at 20 ng/ml (eBioscience) and TGFβ at 1 ng/ml (R&D Systems). The blocking antibodies against cytokines were used at a concentration of 20 µg/ml.

### Cytokine assessment

Sorted cells were incubated in RPMI medium supplemented with 10% FCS, 100 units/ml of penicillin-streptomycin, 2 mM L-glutamine (Gibco), 1 µg/ml ionomycin and 0.01 µg/ml phorbol myristate acetate (PMA; Sigma) for 24 h. Then, supernatants were collected and stored at −80°C until use. Cytokines were evaluated by ELISA (eBioscience), according to manufacturer's protocol.

### ELISpot

The ELISpot assays were performed according to manufacturer's protocol (eBioscience).

### Intracellular cytokine staining

Cells were incubated in RPMI medium supplemented with 10% FCS, 100 units/ml of penicillin-streptomycin, 2 mM L-glutamine (Gibco) in the presence of 1 µg/ml ionomycin and 0.01 µg/ml PMA for 4 h. For the last 2 h of culture Brefeldin A (Sigma) was added to the final concentration 5 µg/ml. After washing, cells were stained with antibodies against surface markers and fixed for 20 min in 2% paraformaldehyde. For intracellular cytokine staining, cells were incubated on ice for 30 min in permeabilization buffer (0.5% saponin and 0.5% bovine serum albumin [BSA] in PBS). After washing, cells were stained with antibodies in permeabilization buffer for further 30 min. Following 2 additional washing steps in permeabilization buffer and one in PBS, cells were analyzed by flow cytometry.

### Real-time PCR

Cells obtained from the *in vitro* proliferation tests (see above) were used for RNA isolation which was performed with the RNeasy Kit from Qiagen, according to manufacturer's protocol. Then, cDNA was generated from these samples using the RevertAid First Strand cDNA Synthesis Kit from Fermentas. For real-time PCR, the SYBR Green PCR Master Mix from Applied Biosystems was used, employing 500 ng of cDNA, and 6 pmol of forward and reverse primers for the genes encoding beta-actin and RORgt (ACTB forward 5′-CCACCGATCCACACAGAGTA-3 and reverse 5′-GGCTCCTAGCACCATGAAGA-3′; and RORC forward 5′-CACAAATTGAAGTGATCCCT-3′ and reverse ′5-AACTTGACAGCATCTCGG-3′, respectively). The samples were run using an Applied Biosystems 7500 Real-Time PCR system. The expression of β-actin was exploited for the quantitation and normalization of the used cDNA. Results for RORC were normalized with respect to the “αGCPEG without NKT cells” condition, which was considered as 100%.

### Statistical analysis

The statistical analysis was performed using the unpaired Student's *t* test. Values of p<0.05 were considered significant.

## Results

### αGCPEG blocks induction of Th17 immune responses after i.n. immunization

**Figure 1:**
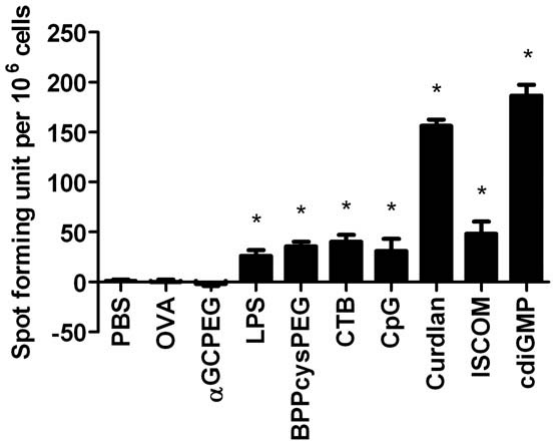
αGCPEG blocks induction of Th17 immune responses after i.n. immunization. C57BL/6 mice were immunized with OVA co-administered with different adjuvants. Control animals received PBS or OVA alone. After 2 boosts on day 14 and 21, splenocytes were isolated and cells were then cultured in ELISpot plates coated with anti-IL-17A antibody for 48 h. Subsequently, plates were incubated with detection antibody, developed and the numbers of spots were counted. From the presented data the background was subtracted. *, statistically significant (p<0.05). Results belong to one representative out of 3 independent experiments.

Our previous studies showed that i.n. immunization leads *per se* to the stimulation of a Th17 immune response [15]. In this work, we immunized animals with OVA co-administered with a broader spectrum of adjuvants to assess if this represents a universal property associated with i.n. immunization or may be affected according to the adjuvant used. The obtained results showed that despite the fact that the number of cells expressing this cytokine differs in absolute terms from case-to-case depending on the adjuvant tested (**Figure 1**), i.n. administration always leads to the development of cells with a Th17 phenotype. The only exception from this rule was what observed in mice which received αGCPEG as adjuvant, since the amount of IL-17A producing cells was similar to those in non vaccinated control animals.

### NKT cells block Th17 differentiation

**Figure 2:**
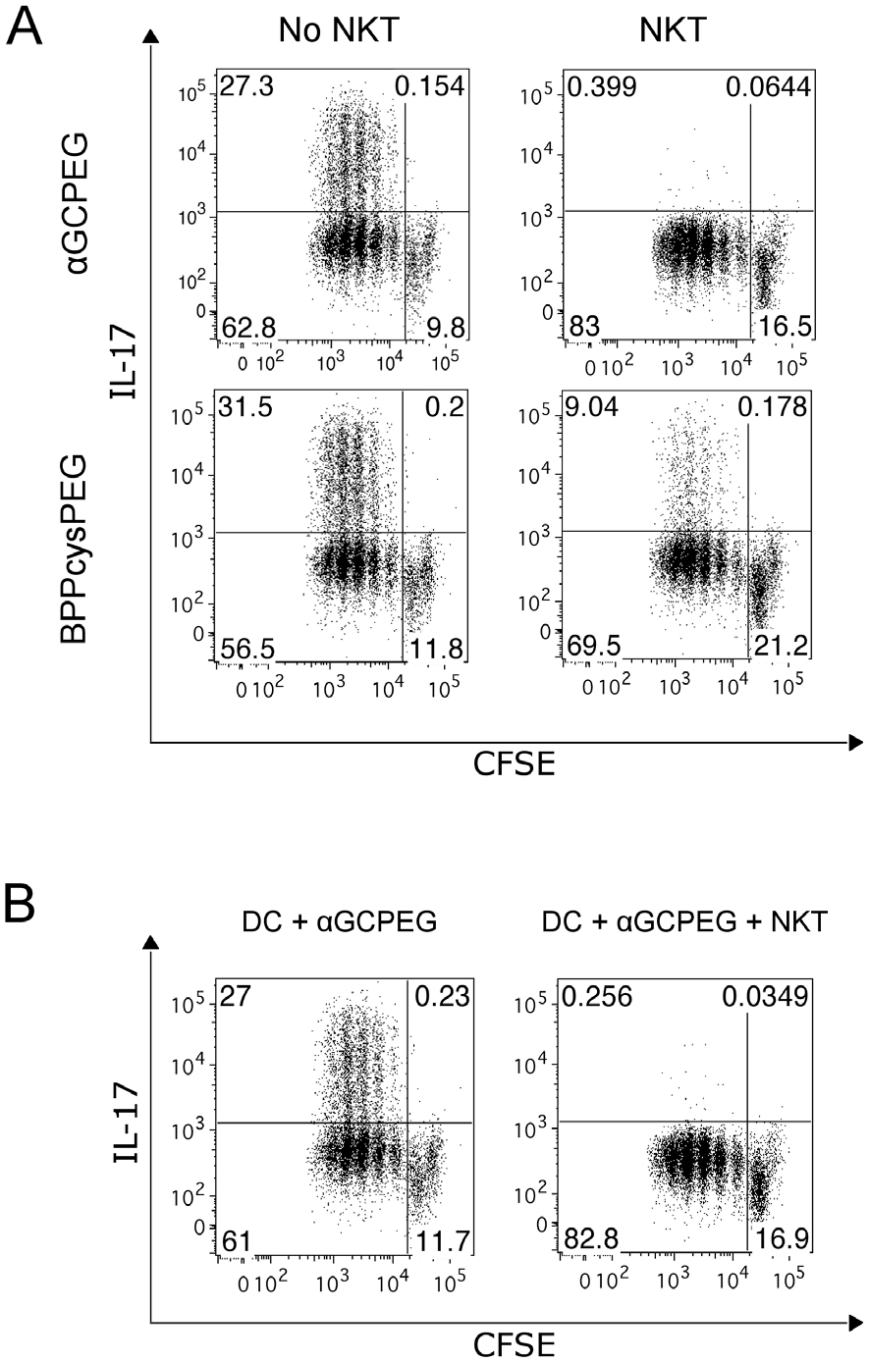
NKT cells block Th17 differentiation *in vitro* by soluble factors. Naïve CD4^+^ cells were sorted from spleen of OTII animals and stained with CFSE. Cells were then co-cultured with DC under Th17 inducing conditions together with a peptide encompassing the specifically recognized epitope (OVA323–339) in the presence of either αGCPEG or BPPcysPEG. After 4 days in culture the cells were stained for IL-17A production and analyzed by FACS. (A) To some cultures NKT cells were added (right panels). (B) To some cultures were added supernatants collected from 24 h cultures of DC in presence of αGCPEG (left panel) or co-cultures of DC and NKT cells in the presence of αGCPEG (right panel). Results belong to one representative out of at least 3 independent experiments.

Considering the mechanisms of action of αGCPEG, we first assessed if NKT cells can block Th17 differentiation. To this end, we stimulated *in vitro* cells from OTII animals with antigen-loaded DC under Th17 inducing conditions in the presence of either BPPcysPEG (a TLR2/6 agonist) or αGCPEG (**Figure 2A**). Under these conditions, OTII cells differentiate in Th17 cells, independently of the stimulator used. Next, we assessed if this basic response pattern was modified in the presence of NKT cells. The obtained results demonstrated that co-incubation with NKT cells results in an almost absolute blockage of IL-17A production when αGCPEG was used as stimulus. We also observed an inhibition of IL-17F production by flow cytometry under these experimental conditions (8–20 fold reduction). In addition, transcriptional analysis by RT-PCR showed a down regulated expression of RORC (5–10 fold reduction) in the presence of NKT cells. The marginal decrease in the percentage of Th17 cells observed in the cultures with BPPcysPEG and NKT cells can be explained by the fact that NKT cells can be activated not only by its cognate ligand, but also by cytokines present in the medium [16].

During the interaction between DC and NKT cells, these cells influence each other by direct and indirect interactions [2]. It was also shown that this interplay results in changes in the global cytokine expression profiles of DC [17]. To evaluate if the observed blockage of Th17 differentiation is due to either a direct interaction between cells or soluble factors, we stimulated *in vitro* cells derived from OTII mice under the above-mentioned Th17 inducing conditions in presence or absence of pre-conditioned supernatants collected from either DC cultures or co-cultures of DC with NKT, which were stimulated with αGCPEG or BPPcysPEG (**Figure 2B**). The results from this experiment showed that the observed blockage is mainly mediated by soluble factors. The presence of DC was necessary for proper activation of NKT cells and obtaining the pre-conditioned medium using αGCPEG as stimulus, since NKT cells are only activated by αGC when it is presented on CD1d molecules (i.e. using DC or tetramer loaded with αGC).

**Figure 3:**
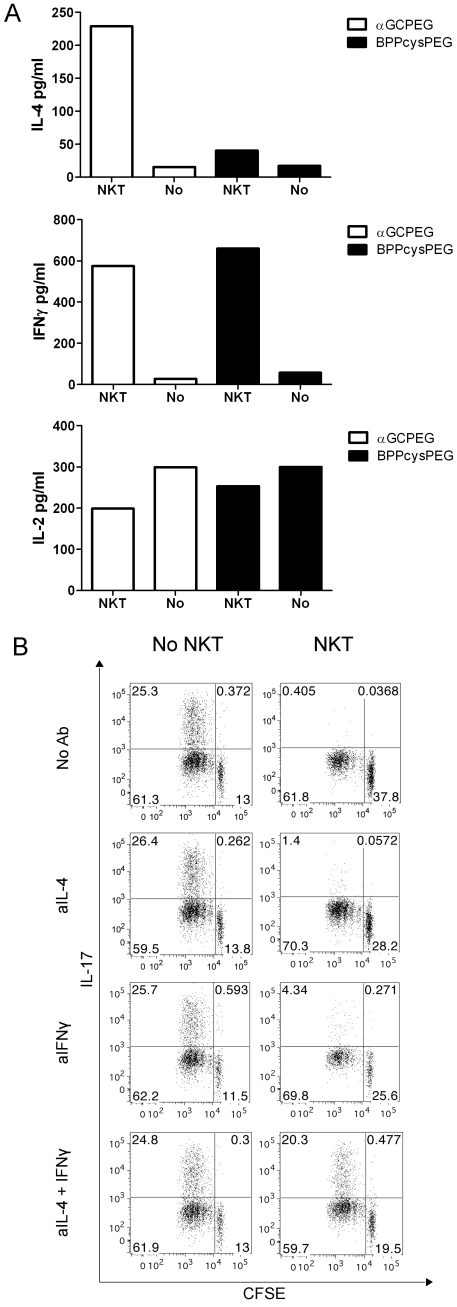
NKT cells block Th17 differentiation by secretion of IL-4 and IFNγ. (A) Naïve CD4^+^ cells were sorted from spleens of OTII animals. Cells were then co-cultured with DC under Th17 inducing conditions with the specific peptide (OVA323–339) under stimulation with either αGCPEG or BPPcysPEG in the presence or absence of NKT cells. After 24 h culture supernatants were collected and the levels of IL-4, IFNγ and IL-2 were measured by ELISA. (B) Naïve CD4^+^ cells were sorted from spleen of OTII animals and stained with CFSE. Cells were then co-cultured with DC under Th17 inducing conditions with the specific peptide (OVA323–339) and αGCPEG, in the presence or absence of NKT cells. To some cultures neutralizing antibodies against IL-4 and/or IFNγ were added. After 4 days of culture the cells were stained for IL-17A production and analyzed by FACS. Results belong to one representative out of at least 3 independent experiments.

NKT cells express different cytokines [2] from which at least IFNγ, IL-4 and IL-2 are known to block Th17 differentiation [18], [19]. To evaluate which of these cytokines might be responsible for the observed blockage of Th17 differentiation, we first assessed their levels in supernatants of co-cultures of OTII cells and DC stimulated with αGCPEG or BPPcysPEG in the presence or absence of NKT cells (**Figure 3A**). The obtained results showed that from the tested candidates IL-2 most probably does not play a role, since the levels of this cytokine were higher in the co-cultures where NKT cells were absent. In contrast, we observed increased levels of both IFNγ and IL-4, most probably produced by NKT cells, as described in the literature [20], [21]. Thus, we then tested if addition of blocking antibodies against these cytokines would result in a restored IL-17A production (**Figure 3B**). The obtained results demonstrated that the presence of blocking antibodies against IFNγ and IL-4 resulted in increased levels of IL-17A production. This effect was even stronger when these two blocking antibodies were combined. Since a previous report indicated that IL-10 might be also involved in inhibition of Th17 development by NKT cells [7], we also evaluated the levels of this cytokine. However, we did not detect IL-10 in the supernatant fluids of co-cultures with OTII cells. Furthermore, addition of blocking antibodies against IL-10 did not result in a reestablishment of IL-17A cell differentiation (data not shown).

**Figure 4:**
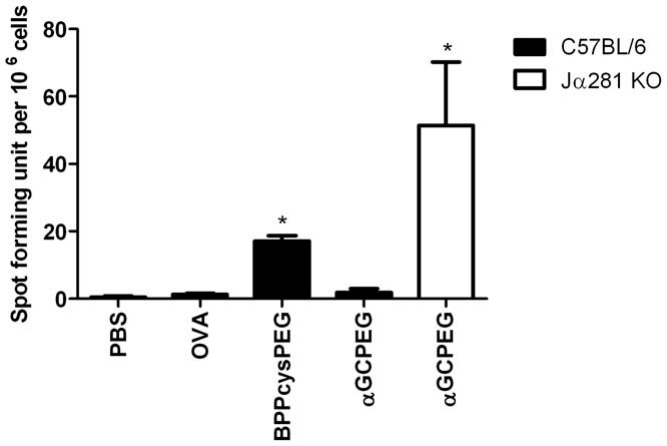
NKT cells block Th17 differentiation after i.n. immunization *in vivo*. C57BL/6 and Jα281 KO mice were immunized with OVA co-administered with αGCPEG. Control animals received PBS, OVA or OVA with the TLR2/6 agonist BPPcysPEG. After 1 boost on day 14, splenocytes were isolated and cells (1×10^6^/well) were then cultured in ELISpot plates coated with anti-IL-17A antibodies for 48 h. Subsequently, plates were incubated with detection antibodies, developed and the numbers of spots were counted. From the presented data the background was subtracted. *, statistically significant (p<0.05). Results belong to one representative out of 2 independent experiments.

To further confirm the role of NKT cells in the inhibition of Th17 differentiation after i.n. immunization with αGCPEG, we performed immunization studies with wild type (WT) mice and Jα281 KO animals, which are deficient for iNKT cells (**Figure 4**). While Th17 cell differentiation was blocked in WT animals receiving the antigen together with αGCPEG by i.n. route, antigen-specific IL-17A producing cells were detected in Jα281 KO animals, which do not have iNKT cells. This confirmed that NKT cells are responsible for the observed αGCPEG-mediated *in vivo* blockage in Th17 polarization.

**Figure 5:**
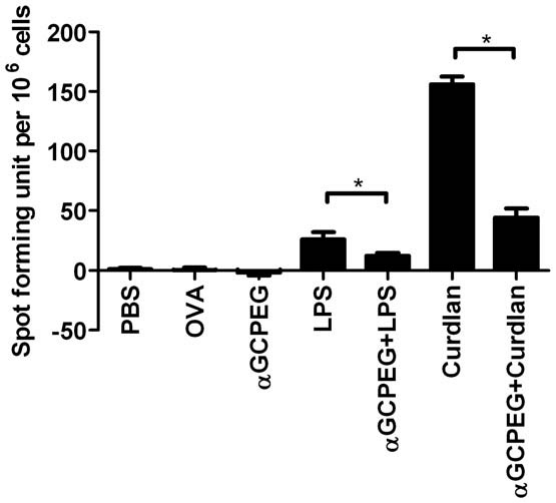
αGCPEG efficiently modulates the effect of other adjuvants. C57BL/6 mice were immunized with OVA co-administered with different combinations of LPS, Curdlan and αGCPEG. Control animals received PBS or OVA alone. After 2 boosts splenocytes were isolated from animals and cells (5×10^5^/well) were then cultured in ELISpot plates coated with anti-IL-17A antibody for 48 h. Subsequently, plates were incubated with detection antibody, developed and the numbers of spots were counted. From the presented data the background was subtracted. *, statistically significant (p<0.05). Results belong to one representative out of 3 independent experiments.

Next, we assessed if αGCPEG can also modulate the effect of other adjuvants. To this end, mice were immunized using as adjuvant LPS or Curdlan alone or co-administrated with αGCPEG (**Figure 5**). The number of cells producing IL-17A was significantly lower in the groups of animals which received αGCPEG than in those immunized with LPS or Curdlan alone. However, the induction of Th17 cells was not completely blocked, as we observed in case of animals immunized with αGCPEG alone. This clearly demonstrates that αGCPEG can be exploited to modulate the biological activities of other adjuvants.

### NK1.1^−^ NKT cells block Th-17 differentiation

**Figure 6:**
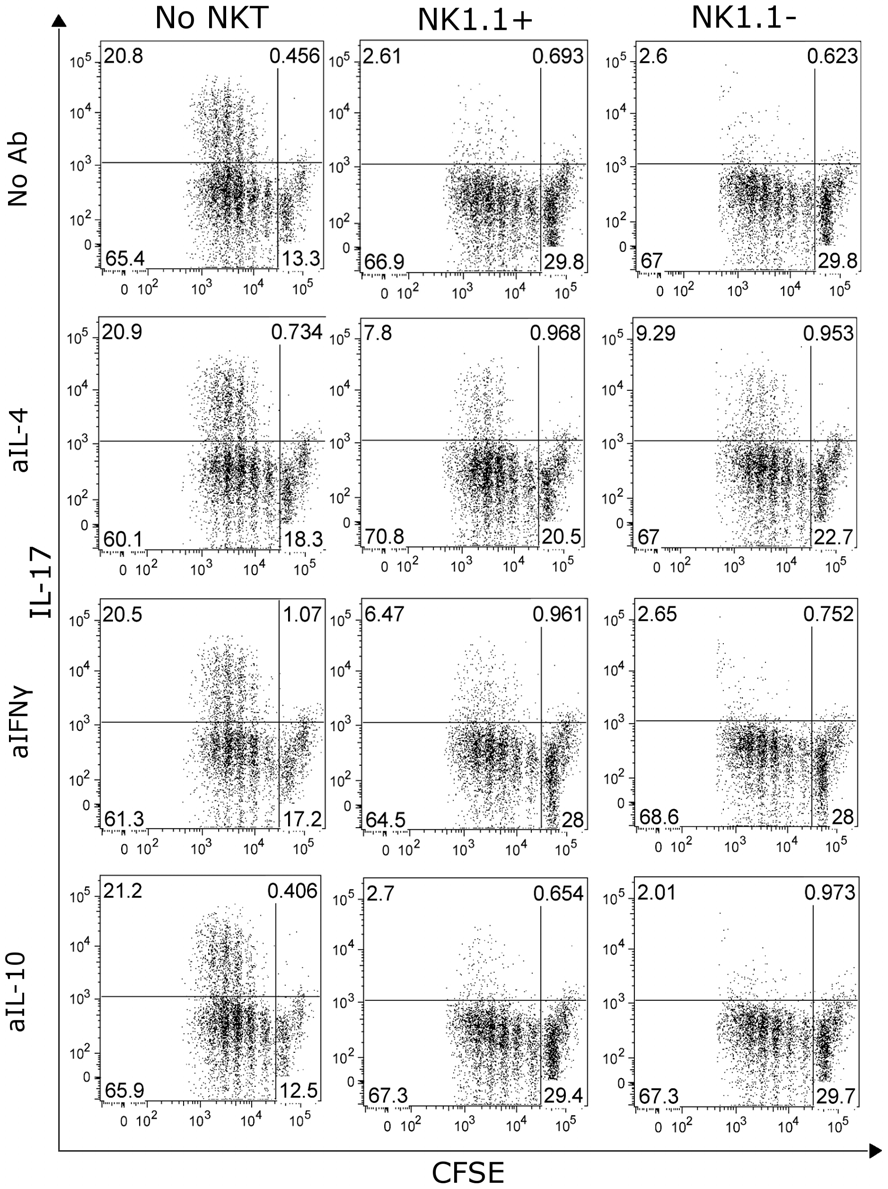
NK1.1^−^ NKT cells also block Th17 differentiation. Naïve CD4^+^ cells were sorted from spleen of OTII animals and stained with CFSE. Cells were then co-cultured with DC under Th17 inducing conditions with the specific peptide (OVA323–339) and αGCPEG in the absence (left panels) or in the presence of either NK1.1^+^ (central panels) or NK1.1^−^ (right panels) NKT cells. Some cultures also received blocking antibodies against IL-4, IL-10 or IFNγ. After 4 days in culture, cells were stained for IL-17A production and analyzed by FACS. Results belong to one representative out of 3 independent experiments.

Several recent reports demonstrated the existence of a special iNKT cell subpopulation, which is characterized by the lack of the NK1.1 molecule and production of IL-17A [9], [10], [11]. This cell subpopulation is present in the respiratory tract of mice. Thus, we wanted to assess if despite their contribution to IL-17A production, these cells can also contribute to the blocking of Th17 cell differentiation. For this purpose we stimulated OTII cells in Th17 inducing conditions alone or in co-culture with NK1.1 positive and negative NKT cells (**Figure 6**). The results from this experiment showed that NK1.1 negative NKT cells also block Th17 differentiation. Since NK1.1 positive and negative NKT cells differ in the production of cytokines other that IL-17A (data not shown), we tested which cytokines are responsible for the blockage of Th17 differentiation in the case of NK11^−^ NKT cells. This was critical, due to the fact that this specific subpopulation was excluded from our previous *in vitro* experiments, since the sorting strategy used for isolation of NKT cells included NK1.1 as a positive marker for these cells. As expected from their cytokine production profile, NK1.1 negative cells mediate this blockage via IL-4, having IFNγ a marginal contribution, if any at all.

## Discussion

The aim of vaccination is to induce adaptive immunity and generate memory cells able to combat a specific pathogen, which might be encountered in the future by the vaccinee. Since an optimal protective immune response against different pathogens requires different Th phenotypes, it follows that it could be beneficial to specifically stimulate appropriate Th cell subsets following vaccination. In this particular context, the stimulation of Th17 cells might be beneficial for clearance of different infectious agents, but IL-17A was also reported to be involved in the pathogenesis of autoimmune diseases [22], [23], [24], [25]. It follows, that it would be extremely helpful to have tools enabling to trigger or block at will the stimulation of antigen-specific Th17 cells.

In our previous studies we showed that i.n. immunization leads *per se* to the induction of a Th17 immune response, which can make in turn this administration route an attractive alternative for certain applications. In the present work we demonstrated that the Th17 phenotype generated by i.n. immunization can be easily manipulated. Our results showed that when an antigen is co-administrated by i.n. route using αGCPEG as adjuvant, the development of Th17 cells is completely blocked. The αGCPEG can be also exploited to modulate the Th17 response pattern triggered by other adjuvants. The blockage of Th17 differentiation is mediated by iNKT cells. This is supported by the results of *in vitro* experiments showing that differentiation of IL-17A producing CD4^+^ cells under Th17 inducing conditions is blocked by NKT cells stimulated with αGCPEG. This was further confirmed by the observed inhibition of IL-17F production and the down-regulated expression of RORC. Preliminary results also suggested that there is a reduction in the production of IL-22 (data not shown).

These results were corroborated by *in vivo* studies in which efficient differentiation of Th17 cells was observed after immunization using αGCPEG as adjuvant in mice lacking iNKT cells (i.e. Jα281 KO animals). Additional *in vitro* studies suggested that this blockage is mediated by IL-4 and IFNγ. Interestingly, despite their capacity to produce IL-17A, NK1.1^−^ NKT cells also block differentiation of Th17 cells. However, in the particular case of this NKT cell subpopulation IL-4 seems to play the major role for blocking Th17 responses.

By and large, our results and those from other groups [7] demonstrate the important role played by NKT cells in the dynamic control of IL-17A expression, especially in those territories where NK1.1 negative NKT cells are present. It can be hypothesized that while significantly contributing to the early production of IL-17A during infection, later these cells can become instrumental for blocking differentiation of Th17 cells. This is especially interesting taking under consideration reports in which a time-dependent role was proposed for IL-17A, implicating early IL-17A production for subsequent Th1 immunity [26].

In conclusion, our results have demonstrated that αGCPEG can be considered as the adjuvant of choice for clinical applications in which it is desired to combine the benefits of i.n. immunization with the stimulation of Th phenotypes other than Th17. In this context, αGCPEG usually leads to Th2 dominant immune response, as demonstrated by previous studies from us [14] and others [3]. This is independent of either the antigen evaluated or the haplotype of the tested mouse strain. However, there are other compounds recognized by TCR of iNKT cells, which due to differential affinity to invariant TCR lead to Th1 immune responses [2], [27]. The use of these compounds might represent a valid strategy to modulate Th responses by using a single agonist of the TCR expressed by iNKT cells. On the other hand, we showed that co-administration of αGCPEG with other adjuvants also results in a significantly reduction in the number of IL-17A producing memory cells. This shows that αGCPEG can be easily exploited to modulate responses stimulated by other adjuvants, thereby dramatically expanding its potential applications for fine-tuning immune responses to vaccines.
